# Sensitivity of planktic foraminiferal test bulk density to ocean acidification

**DOI:** 10.1038/s41598-019-46041-x

**Published:** 2019-07-05

**Authors:** S. Iwasaki, K. Kimoto, O. Sasaki, H. Kano, H. Uchida

**Affiliations:** 1 Research Institute for Global Change, JAMSTEC, 2-15 Natsushima-cho, Yokosuka, 237-0061 Japan; 2 The Tohoku University Museum, Tohoku University, 6-3 Aoba, Aramaki, Aoba-ku, Sendai, 980-8578 Japan

**Keywords:** Carbon cycle, Palaeoceanography, Marine biology

## Abstract

The anthropogenic CO_2_ accumulating in the ocean is lowering seawater carbonate ion concentration and may reduce calcification rates of marine calcareous organisms. Several proxies based on test weights of planktic foraminifera have been used to evaluate the impact of ocean acidification on these organisms. Unfortunately, because of the absence of a method to evaluate the bulk density of a test, the impact of seawater carbonate chemistry on test calcification is still not fully understood. In this study, we measured bulk densities of living *Globigerina bulloides* (planktic foraminifera) tests with an X-ray micro-computed tomography (XMCT) scanner and compared them with ambient seawater characteristics. Results demonstrated that test bulk densities were controlled by ambient seawater carbonate ion concentrations and that changes of test bulk densities were accompanied by changes in micron to submicron scale porosity of internal ultrastructure. These results suggest that alteration of the bulk density of foraminiferal tests due to acidification of ambient seawater can be directly observed by XMCT scanning. A useful metric of calcification intensity would therefore be physical measurements of test densities with XMCT.

## Introduction

Some of the CO_2_ gas discharged by human activities is being absorbed into ocean surface waters, where it is causing a decrease of seawater pH and concomitant reduction of the seawater carbonate ion concentration, [CO_3_^2−^]. The latter effect is adversely impacting the production of calcium carbonate by marine organisms (e.g., coccolithophores, foraminifera, and pteropoda)^1^. Acidification of the surface and subsurface waters of the western subarctic Pacific has been clearly documented at two stations (Station K2 and KNOT) over the past decade; the pH of the surface water in that area has changed at a rate of −0.0011 ± 0.0004 per year from 1997 to 2011 (∼0.015 decrease in pH during that time), a rate consistent with the rate of increase of atmospheric *p*CO_2_ ^2^.

Planktic foraminifera dwell throughout the ocean, and their tests are major components of ocean carbonate sediments^3^. Investigations of the impact of ocean acidification on the production of carbonate tests by planktic foraminifera will enable a more informed assessment of the impact of ocean acidification on open ocean ecosystems. Furthermore, investigation of how the effect of the seawater condition is recorded in the physical characteristics of foraminiferal tests is beneficial to improving the accuracy of paleoceanographic proxies. A number of studies have investigated the relationship between the intensity of foraminiferal test calcification and sea water chemistry by using metrics of size-normalized weight (SNW) such as sieve-based weight (SBW), which is the mean bulk weight of tests from a narrow size fraction^4–6^, and measurement-based weight (MBW), which reduces the influence of test size on the weight measurement^7–9^. The studies using these proxies pointed out that foraminiferal shell weight is controlled by various factors of sea water condition depending on species based on the records of fossil in sediment samples across latitude or glacial-interglacial. Furthermore, the test area density (µg/µm^2^), which is the ratio of the weight (µg) of a test to its projected area (µm^2^), is a more rigorous size-normalized proxy and considered to be an effective indicator of the calcification intensity of foraminiferal tests^10^. For example, reductions of planktic foraminiferal test area density with decreasing seawater [CO_3_^2−^] have been reported in sediment trap samples, and test area density has been suggested for use as a reliable proxy for sea surface [CO_3_^2−^]^10,11^. The size normalized test weight proxies, including test area density, is considered to represent the test wall thickness and that recognized as calcification intensity of foraminifera^7,10^. However, because the test area density is normalized by 2-D projection area observed externally, it does not provide an evaluation of conditions inside the test. The bulk density of a test, on the other hand, is one of its important indications of test condition of foraminifera that is likely to be controlled by seawater carbonate chemistry. In this study, the bulk density (g/cm^3^) of test is defined as the ratio of weight to volume of individual test calcite. It is affected not from test wall thickness but from micron to submicron scale porosity in internal ultrastructure of test calcite. In the previous studies, however, there have been no methods for quantifying the bulk density of individual foraminiferal tests, and thus impact of seawater condition to the bulk density of foraminiferal test is not understood. New methods for quantifying the bulk density of test are required to understand the impact of ambient carbonate chemistry on foraminiferal test calcification.

Progress in X-ray micro-computed tomography (XMCT) has allowed researchers to observe the three-dimensional (3-D) skeletal structures of microorganisms. In recent studies, XMCT scanning has been used to observe the internal test structure of planktic foraminifera^12,13^. In addition to providing a 3-D image, XMCT facilitates quantitative estimation of the volume of a foraminiferal test, which enable us to calculate the bulk density (g/cm^3^) of individual test. Furthermore, the computed tomography (CT) number is calculated from X-ray coefficient, which is affected by micron to submicron scale porosity in ultrastructure of test calcite, and is a suitable metrics to quantify not only the bulk density of whole test but also density of specific part of calcite in individual test (e.g., density chamber by chamber). Thus, we consider that XMCT scanning is a method well suited for quantifying test condition of foraminifera from the view point of test bulk density unaffected by test wall thickness and could supplement or even replace information on test weights in studies of ocean acidification effects.

**Figure 1:**
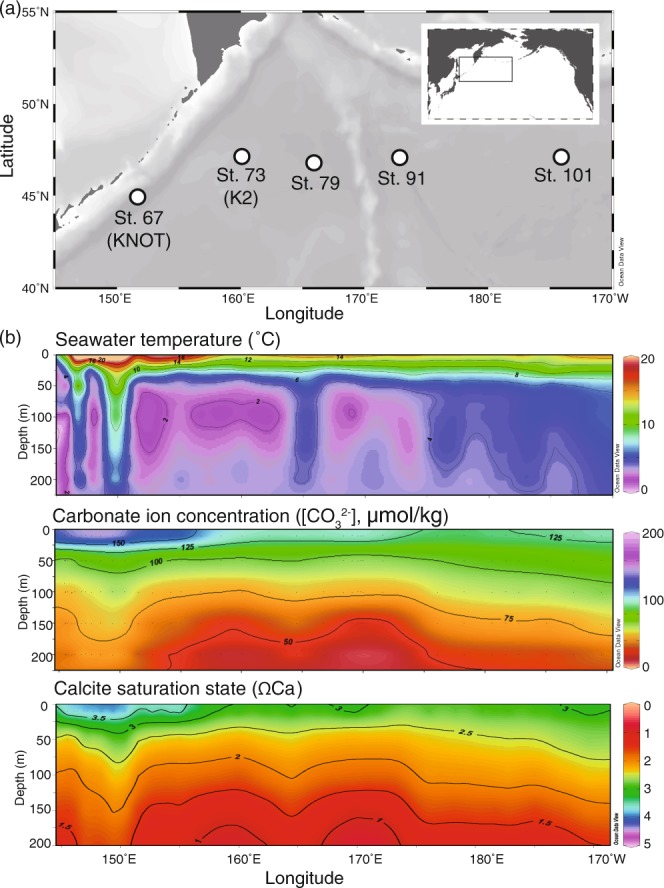
(**a**) Location of sampling stations in the subarctic North Pacific. White circles indicate stations where plankton tows were conducted during leg 2 of cruise MR14-04 of the R/V Mirai. (**b**) Depth–latitude sections of seawater temperature (°C), carbonate ion concentration [CO_3_^2−^] (µmol/kg) and saturation state with respect to calcite (ΩCa) in the study area. Map and figures were drawn by Ocean Data View (ODV)^25^.

To understand the impact of ambient seawater conditions on the physical characteristics of tests of live foraminifera during calcification, we studied variations of the characteristics of tests of live planktic foraminifera (*Globigerina bulloides*) collected via plankton tows at five sites located along the WOCE-P01 line, one of the survey lines of the World Ocean Circulation Experiment (WOCE) in the subarctic North Pacific (Fig. 1). We used XMCT scanning and test weight measurements to quantify the physical characteristics of the tests (wall thickness, test bulk density, and area density) of live *G. bulloides* and compared them with the characteristics of ambient seawater where the samples were collected (Supplementary Table S1). Such information is expected to facilitate the understanding of sea water chemistry effect to the calcification of planktic foraminifera.

## Results

### Relationship between ambient seawater characteristics and the physical characteristics of *G. bulloides* tests

**Figure 2:**
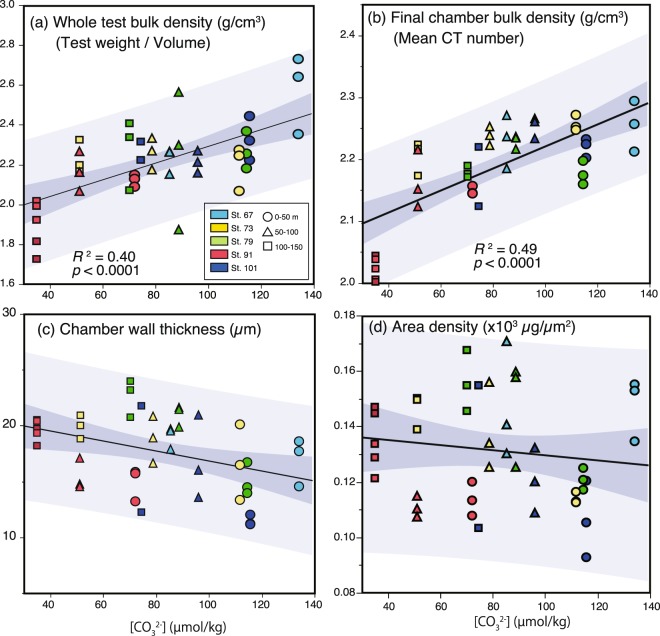
Plots of (**a**) whole test bulk density (g/cm^3^) based on individual shell weight and volume measurement, (**b**) final chamber test bulk density (g/cm^3^) based on mean CT number, (**c**) Outermost chamber wall thickness (µm), and (**d**) the area density (µg/µm^2^) of individual *G. bulloides* tests against ambient seawater [CO_3_^2−^] (µmol/kg) at each water depth of sampling sites of plankton tow pobservation. Sample sites and depths are indicated by colors and symbols, respectively. The coefficient of determination (*R*^2^), *p*-value, regression line, and the 95% confidence interval (dark shading) and prediction interval (light shading) are shown in each plot.

Quantitative measurements of physical characteristics of live *G. bulloides* test (whole test bulk density, final chamber bulk density, mean wall thickness, and area density), which were sampled from seawater supersaturated with calcite (ΩCa > 1), showed which physical characteristics of the tests were most sensitive to ambient seawater [CO_3_^2−^] where they sampled (Fig. 2). Ambient [CO_3_^2−^] values were more significantly correlated with the whole and final chamber test bulk densities (g/cm^3^) than with the mean wall thickness (µm) and area density (µg/µm^2^) of the tests (Fig. 2a,b). In particular, the test bulk densities of the final chambers of each *G. bulloides*, calculated by mean CT number, were the most significantly correlated (*p* < 0.0001) with ambient seawater [CO_3_^2−^]; this density was around 2.25 g/cm^3^ when ambient seawater [CO_3_^2−^] exceeded 130 µmol/kg and around 2.00 g/cm^3^ when ambient seawater [CO_3_^2−^] was less than 40 µmol/kg. The results of multiple regression analysis between proxies of physical characteristics of test and parameters of ambient seawater condition where they sampled (response variable: proxy of physical characteristics; explanatory variables: parameter of ambient seawater condition; temperature, salinity, concentrations of chlorophyll-a, nitrate, and [CO_3_^2−^]) suggested that both whole test and final chamber bulk density was the most sensitive to ambient seawater [CO_3_^2−^] (Supplementary Table S2). On the other hand, the wall thickness and area density of *G. bulloides* tests were not correlated with ambient seawater [CO_3_^2−^] (Fig. 2b,c), and results of multiple regression analysis showed that these proxies receive relatively large contribution from ambient seawater temperature. However, because seawater temperature decreases with depth, these results seem to suggest the increase of test wall thickness and area density with deepening of water column.

### Changes of test cross-sectional ultrastructure

**Figure 3:**
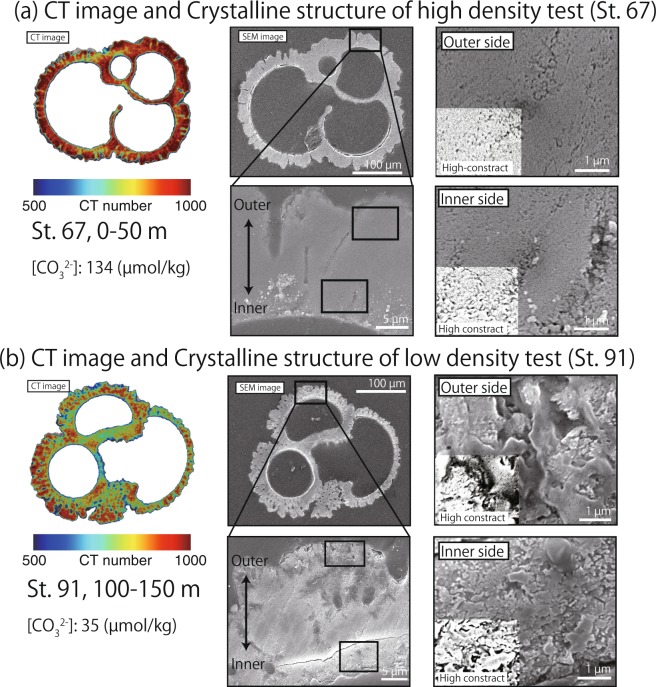
Comparisons between CT and SEM images of *G. bulloides* tests (same individual), the densities of which were (**a**) high (obtained from St. 67, 0–50 m) and (**b**) low (obtained from St. 91, 100–150 m). SEM images of the cross section of the outermost chamber of *G. bulloides* show the ultrastructure of the outer and inner sides of each test. High-contrast photos of cross section ultrastructure are shown to emphasize the shadow area, indicating the amount of airspace in the test ultrastructure. The higher amount of airspace suggests the higher porosity of ultrastructure in test, which correspond to low CT number area in CT image.

The combined results of scanning electron microscopy (SEM) and CT observations revealed differences between the internal ultrastructure of the test walls that were related to the mean CT numbers of the tests (Fig. 3). High-contrast photos of a part of SEM images are shown to emphasize the shadow area (black area). This shadow area represent the micron to submicron airspace in test calcite and can be recognized as porosity of ultrastructure. High-density tests were obtained from depths of 0–50 m at St. 67, where ambient seawater [CO_3_^2−^] was high (134 µmol/kg). Those tests had high mean CT numbers; a compact, micro-granular crystalline structure on the inner side; and a euhedral crystalline structure with fully grown crystal grains on the outer side. From the high-contrast photos in SEM images, we found small area of airspace (black area), which indicates the low porosity of calcite on both inner and outer side of test wall (Fig. 3a). On the other hand, low-density tests were obtained from depths of 100–150 m at St. 91, where ambient seawater [CO_3_^2−^] was low (35 µmol/kg). Those tests had low mean CT numbers and were characterized by grains and coarse crystalline structures that were larger and more porous, respectively, on both the inner and outer sides than the high-density tests. In addition, high porosity of calcite is also represented by large area of airspace (black area) in high-contrast photos of SEM images (Fig. 3b).

### Changes in the CT number histogram

**Figure 4:**
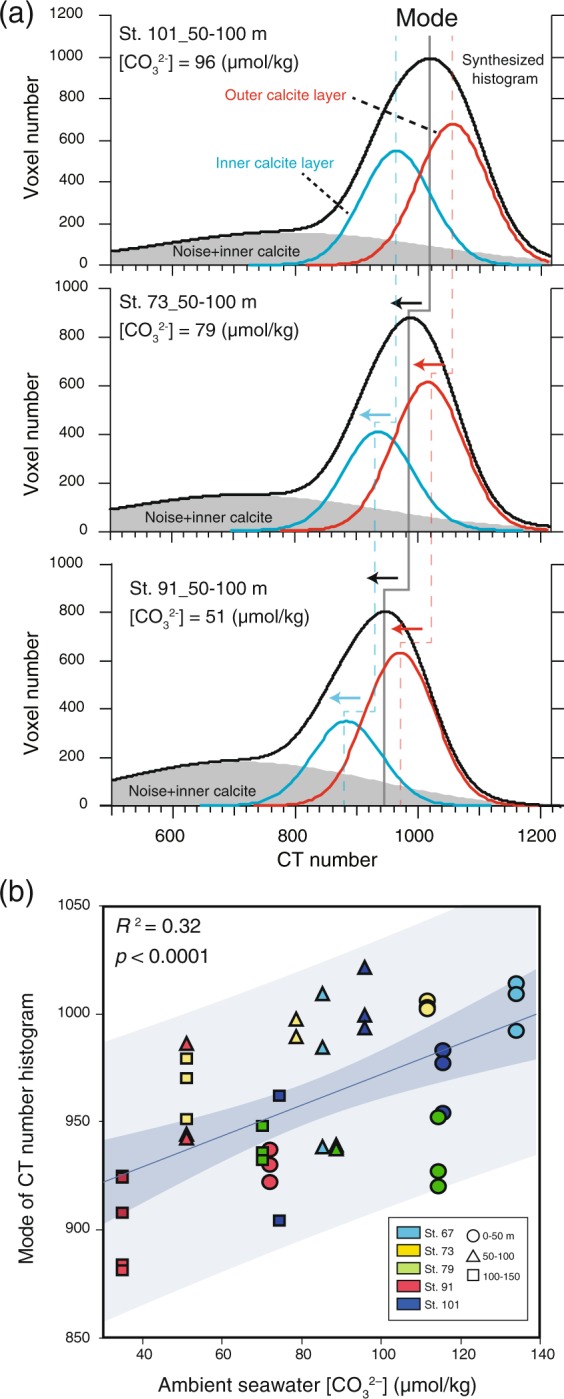
(**a**) Schematic diagrams of *G. bulloides* calcite density distribution changes based on histograms of CT numbers. Change in mode of CT number histogram (black line) along with ambient seawater [CO_3_^2−^] decrease are shown, and fitting of three Gaussian functions: outer calcite layer (red line), inner calcite layer (blue line), and voxels that were assumed to be mainly noise, including a small amount of inner calcite (gray area) to CT number histogram are also shown. Plots of (**b**) mode of CT number histogram against ambient seawater [CO_3_^2−^] (µmol/kg) are shown.

The CT number histograms of *G. bulloides* tests sampled from different ambient seawater [CO_3_^2−^] were characterized by differences in the distributions of calcite density (Fig. 4a). Although all CT number histograms were unimodal under every sampling site condition, shifts of CT numbers to lower values (to the left) were observed as ambient seawater [CO_3_^2−^] decreased. These shifts of CT number are characterized by gradual reduction in the mode of CT number histogram (Fig. 4a, Synthesized histogram), and the mode of CT number histogram showed week positive correlation with ambient seawater [CO_3_^2−^] where they sampled (*R*^2^ = 0.32, *p* < 0.0001) (Fig. 4b). Furthermore, the multiple regression analysis also suggested that the mode of CT number histogram was the most sensitive to ambient seawater [CO_3_^2−^] (Supplementary Table S2). This sensitivity suggests that the reduction in the bulk density of *G. bulloides* test due to ambient seawater [CO_3_^2−^] is characterized by shift of CT number of entire test to lower values.

## Discussion

This study provided an opportunity to examine the relationships between the individual physical characteristics (wall thickness and bulk density) of tests of *G. bulloides* determined via XMCT scanning and ambient seawater characteristics (temperature, salinity, and concentrations of chlorophyll-a, nitrate, and carbonate ions). Linear regression and multiple regression analyses using data of individual test indicate that among ambient seawater characteristics examined in this study, the bulk density of *G. bulloides* tests was positively correlated only with ambient seawater [CO_3_^2−^]. Furthermore, examination of the cross-sectional ultrastructure of the tests via SEM revealed that when ambient seawater [CO_3_^2−^] was less than ~40 µmol/kg, the tests of *G. bulloides* were composed of relatively porous internal ultrastructure. The bulk densities of the tests were thus significantly lower than the bulk densities of tests produced at higher [CO_3_^2−^]. The implications are that a decrease of ambient seawater [CO_3_^2−^] might affect the micron to submicron scale internal ultrastructure of *G. bulloides* tests, and that changes in the porosity of tests due to the impact of reductions of ambient seawater [CO_3_^2−^] could be identified via XMCT scanning. The calcite of foraminiferal tests is thought to be deposited in an organic layer called the Primary Organic Sheet (POS) through uptake of Ca^2+^, CO_3_^2−^, and other ions from the surrounding seawater^14–16^. Furthermore, the pH is thought to be higher in the POS (calcification site) than in the surrounding seawater because of proton pumping during calcification^17^. Based on this scenario, we hypothesize that reduction of ambient seawater [CO_3_^2−^], accompanied by a decrease of ΩCa and pH, may indirectly influence the calcification of foraminifera due to, for example, a relative decline of pH or consumption of energy to raise the pH at the calcification site.

A fundamental ontogenetic process of *G. bulloides* related to its survival (e.g., maintenance of a large population or competition with other species) could be another factor responsible for the reduction of the bulk density of *G. bulloides* tests. An analysis of the same sample dataset used in this study showed a population (standing stock of shells: shells m^−3^) of *G. bulloides* more than three times as large and a larger mean test size at St. 91 than at the other sites^18^. The seawater at St. 91 is characterized by a deeper pycnocline (39 m) and hence greater input of nutrient-rich water from below compared to the other sites. This difference of seawater characteristics indicates that conditions at St. 91 are relatively favorable for photosynthesis because allochthonous nutrient inputs are greater but less favorable for calcification of foraminifera because ambient seawater [CO_3_^2−^] is lower. Because calcification in planktic foraminifera is an energy-consuming process, foraminifera must use energy-efficient strategies to thrive. The co-occurrence of a large population and large tests therefore appears to be inconsistent with a strategy of energy efficiency^18^. Measurement of test bulk densities by XMCT revealed that the bulk densities of *G. bulloides* tests from subsurface (100–150 m) water at St. 91 were low compared to those from other stations. This observation suggests that *G. bulloides* can produce tests with porous crystalline structure to achieve rapid growth (metabolism) and high productivity (standing stock), and furthermore, that seawater with a low ambient [CO_3_^2−^] can cause *G. bulloides* to produce tests with low bulk densities.

To identify the controlling factor of test area density in this study, relationship between individual test physical characteristics (Chamber wall thickness and test bulk density) and individual test area density were observed (Supplementary Fig. S1), and revealed that thickness of outermost chamber wall is a principle factor controlling test area density (Supplementary Fig. S1a, r2 = 0.48, *p* < 0.0001), as already suggested in the previous studies. On the other hand, we suppose that variation in test bulk density is not sufficient to alter the test area density of *G. bulloides* compare to variation in wall thickness in the case of this study. The test area density of *G. bulloides*, which has been recognized as a seawater [CO_3_^2−^] proxy in the previous study, did not correlate with ambient seawater [CO_3_^2−^] in the case of using live *G. bulloides* test obtained by plankton tow in this study. This disagreement was probably caused by secondary thickening of the test wall with deepening and growth of *G. bulloides*, called as “calcite crust”, prior to gametogenesis^19^. In generally, encrusted *G. bulloides* tests have smaller 2-D projection area and thicker test wall compare with non-encrusted test, and thus the test area density of encrusted *G. bulloides* is higher than that of non-encrusted^11^. Although we did not distinguish encrusted and non-encrusted tests in this study due to the difficulty of visual distinction, the plot of test area density versus Size Normalized Shell Weight (SNSW), which is an indicator to differentiate the encrusted and non-encrusted test and each type of tests can be plotted on the different regression lines^11^, showed that all *G. bulloides* tests were plotted on a regression line. (Supplementary Fig. S3). This suggests that live *G. bulloides* tests of this study cannot be divided into encrusted or non-encrusted tests precisely. In the results of this study, on the other hand, both the outermost chamber wall thickness and the area density of *G. bulloides* test obtained from depth transect plankton tow sampling showed approximately 20% increase with deepening of depth from 0–50 to 100–150 m (Supplementary Fig. S2), implying that *G. bulloides* test from deeper layer are much thicker and heavy test than from shallower layer. Based on the above results, we consider that though live *G. bulloides* tests of this study cannot be divided into encrusted or non-encrusted tests precisely, they are under the process of gradual thickening of their outermost chamber with growth. If so, our live *G. bulloides* test sample consists of tests in various growth stage, and variance of outermost chamber wall thickness may have large impact on their test area density. Therefore, we suppose that comparison between test area density of live *G. bulloides* and ambient seawater [CO_3_^2−^] in this study does not make much sense to assess the influence of seawater condition to the calcification intensity of *G. bulloides* test. In other word, the proxy of test area density of *G. bulloides* should be applied into matured individual. The test bulk density measured by XMCT scanning, on the other hand, showed significant correlation with ambient seawater [CO_3_^2−^] (Fig. 2). Furthermore, this correlation is also found even if under the similar thickness of outermost chamber wall (i.e. similar growth stage) (Supplementary Fig. S4). This implies that ambient seawater [CO_3_^2−^] affect the bulk density of live *G. bulloides* test during calcification regardless of difference in their growth stages. However, because *G. bulloides* tests used in this study are supposed to be not full-encrusted, the impact of ambient seawater [CO_3_^2−^] to the bulk density of full-encrusted (matured) test should be examined in the future.

The effect of inorganic dissolution during settlement and at deep sea floor is another principal factor that decrease the weight of foraminiferal test. Because the foraminiferal test dissolution is characterized by selective dissolution of inner chamber wall^12^, the area density of test is substantially influenced by dissolution. Furthermore, study of *G. bulloides* test dissolution process using by XMCT scanning revealed that the bulk density (shown by mean CT number) of *G. bullides* test also decrease with the progression of dissolution as like as the test area density^13^. These results suggested that the proxies of both area density and bulk density of *G. bulloides* test are principally affected by two different factors that are ambient seawater condition during calcification and test dissolution at deep sea floor after settlement. Thus, in order to apply these proxies into the foraminiferal tests from sediment samples in the paleoceanographic study, it has been necessary to eliminate the effect of either factor by using non-dissolved tests in sediment samples above the lysocline or analysis combined with other geochemical proxies. The CT number histogram obtained by XMCT scanning provide a valuable clue to assess the 3-D density distribution in the individual test. A previous study based on observations of test internal ultrastructure by XMCT scanning and SEM analysis has suggested that the outermost chamber of a *G. bulloides* test has a two-layered structure: the outer calcite layer with dense calcite and dissolution resistant, and the inner calcite layer with porous calcite and dissolution prone^13^. The process of dissolution of a *G. bulloides* test is characterized by a change in the CT number histogram from a unimodal distribution with a single high CT number peak to a bimodal distribution with two peaks, which imply the selective dissolution of inner calcite layer. On the other hand, the results of this study suggest that the mode of CT number histogram of a *G. bulloides* test decreased with the reduction in ambient seawater [CO_3_^2−^], but the histogram remained unimodal. This result indicates that process of change in CT number histogram due to the effect of ambient seawater [CO_3_^2−^] during calcification is different from that due to the effect of calcite dissolution at the deep sea floor. As the results of peak fitting of gaussian functions of inner and outer calcite layer to CT number histogram, we found gradual reduction in modes of both functions, the suggestion being that the effect of ambient seawater [CO_3_^2−^] contributes to the reduction in the density of whole *G. bulloides* test (both inner and outer calcite), which is a different characteristics from dissolution process of test at deep seafloor. Here we hypothesize that the CT number histogram of a *G. bulloides* test can provide a useful means for distinguishing the effects of calcification during growth and dissolution after settlement. For example, because the outer calcite layer is supposed to be affected by ambient seawater [CO_3_^2−^] during calcification but highly resistance to dissolution, the mode of the Gaussian function of the outer calcite is possible to be a clue for distinguishing each factors. However, because the data of live *G. bulloides* tests in this study from plankton tow shows snapshot of test characteristics under the several growth conditions and they are supposed to be not full-encrusted (matured) test, there are still a number of issues regarding application of above idea using CT number histogram into the fossil test in the sediment sample.

Our findings based on XMCT scanning and SEM observations of *G. bulloides* tests enabled us to identify the changes in the physical characteristics of foraminiferal tests caused by changes in ambient seawater [CO_3_^2−^] during calcification. Among the physical characteristics of tests, the bulk density, which represents porosity of test ultrastructure and can be measured by XMCT scanning, is the most sensitive to ambient seawater [CO_3_^2−^] where tests calcified. This suggests that reduction in ambient seawater [CO_3_^2−^] affect the calcification system of *G. bulloides* and promote the formation of porous test ultrastructure. Therefore, the test bulk density using by XMCT scanning is supposed to be a suitable marker for assessing the impact of ambient seawater acidification to the ontogenetic calcification of live *G. bulloides*. In addition, our results revealed that the thickness of outermost chamber is a principal factor controlling the proxy of area density of *G. bulloides* test, which implies that ontogenetic growth stage of *G. bulloides* is one of the important factor for the area density in the case of investigation in live *G. bulloides*. Quantification of calcareous test bulk density via XMCT scanning can potentially be applied to calcareous organisms other than planktic foraminifera and will enable quantitative comparisons of test densities between organisms. Direct measurement of test bulk densities by XMCT scanning will undoubtedly be a useful method for monitoring and quantifying the impact of ocean acidification on planktic foraminifera and other calcareous organisms and will be an indispensable tool in the field of ocean acidification research.

## Methods

### The foraminiferal samples

We studied *Globigerina bulloides*, one of the most abundant species of planktic foraminifera in the western North Pacific^20,21^. Because the internal structure of tests of *G. bulloides* has been thoroughly investigated with an XMCT scanner^13^, it was possible to identify changes of the test structure due to differences in growth conditions. The tests of *G. bulloides* were collected by vertical tows of a messenger-released plankton net (frame size: 0.16 m^2^) at five sites in the subarctic Pacific Ocean from late July 2014 to late August 2014 during leg 2 of cruise MR14-04 of the R/V Mirai as a part of the Post–WOCE Hydrography project (Fig. 1). The mesh size of the net was 63 µm, and the sampling depth ranges were 0–50, 50–100, and 100–150 m. The collected samples were sieved with a 45-µm mesh nylon net, and the residue retained on each net was washed into a sample cup with ethanol and preserved in a refrigerator at 3 °C. In the laboratory, all planktic foraminiferal tests larger than 100 µm were collected from each sample cup^18^. Before carrying out the picking, we determined that most individuals were alive by visually confirming that most foraminiferal shells contained cytoplasm. After the collected tests had been dried, the *G. bulloides* tests in the size range 200–350 µm were picked under a dissecting microscope (SZX-7, OLYMPUS, Tokyo, Japan) for subsequent analysis. Supplementary Table S1 provides a summary of the station locations, sample depths, number of samples, and the characteristics of the seawater from which the tests were collected.

### Area-normalized shell weight (Area density) measurements

We calculated size-normalized weights of *G. bulloides* tests based on the method of area density^10^. The weights of individual *G. bulloides* tests in the size range 200–350 µm that were picked from the plankton tow samples were measured with an ultra-microbalance (Cahn C-35, Thermo Electron Corp., Round Rock, TX, USA). The analytical precision of the weight measurement was ±0.3 µg (±1 σ) based on 15 repeated measurements of a single specimen. After the measurements of shell weights, the 2-D projected area (µm^2^) of individual tests was measured with image analysis software (Motic Image Plus 2.1S, Shimadzu Rika Corp., Tokyo, Japan). Area densities of *G. bulloides* tests were calculated using the following equation^10^:

$${\rm{Area}}\,{\rm{density}}\,({\mu g/\mu {\rm{m}}}^{2})={\rm{individual}}\,{\rm{weight}}\,(\mu g)/{\rm{individual}}\,{\rm{projected}}\,{\rm{area}}\,({\mu {\rm{m}}}^{2})$$

### X-ray micro-CT scanning

An XMCT system (ScanXmate-D160TSS105/11000, Comscantecno Co., Ltd., Yokohama, Japan) in the Tohoku University Museum was used to determine the bulk density and wall thickness of foraminiferal tests. A high-resolution setting (X-ray focus spot diameter of 0.8 µm, X-ray tube voltage of 80 kV, detector array size of 2000 × 1336, 1500 projections/360°, 2.5 s/projection) was used for 3-D quantitative densitometry of the small foraminiferal tests. Details of the scanning conditions and of the method used to normalize the CT number were based on a previous study^13^. A particle of limestone (NBS 19; ~130 µm in diameter; 2.71 g/cm^3^ in true density; 1000 in mean CT number) was used to normalize the CT number of foraminiferal tests. The analytical precision of the mean CT number was ±13 (±1 σ) based on five repeated measurements. To eliminate the influence of artifacts due to beam hardening effects, we compared results obtained with a similar XMCT system in the Japan Agency for Marine-Earth Science and Technology, which had been modified to remove beam-hardening effects. Fifteen specimens were selected from a sample set and scanned by both XMCT systems, and the mean CT number of the tests in this study were revised to cancel the influence of the beam hardening effect. From the 3-D scanning data of foraminiferal test, we obtained CT number of each voxel and volume (µm^3^) of each individual test. We used the following proxies to estimate the physical characteristics of the foraminiferal tests in addition to the area density: (1) Whole test bulk density (g/cm^3^), which is defined as the ratio of weight to volume of individual test; (2) Final chamber bulk density (g/cm^3^) based on mean CT number of final chamber calcite for the investigation of the direct impact on test calcification of the characteristics of ambient seawater where the tests were collected; (3) Wall thickness (µm) of outermost chambers, which is average thickness of four chambers from final measured on isosurface CT image; and (4) the CT number histogram of tests for the evaluation of micron to submicron scale density distribution in the individual test. To quantify the changes in the histogram and obtain a consolidated understanding of the condition of live *G. bulloides* tests, we analyzed the shapes of the CT number histograms by fitting the data to three Gaussian functions (outer calcite layer with high CT number, inner calcite layer with lower CT number, and the noise area [e.g., artifact at air or boundary between test and air], including a small amount of the inner calcite) with IGOR Pro software (WaveMetrics, Inc., Lake Oswego, OR, USA).

### Translation from mean CT number to bulk density of foraminiferal test

A regression equation was used to convert the mean CT number of test calcite to the bulk density (g/cm^3^) of the test. Ten specimens were randomly selected from a sample set, and the bulk densities of individual tests were estimated by dividing the test volumes (measured by XMCT) by the test weights (measured with an ultra-micro balance). The analytical precision of the test bulk density was ±0.1 g/cm^3^ (±1 σ) based on 10 repeated measurements. The regression equation describing the relationship between the mean CT number and the bulk density of foraminiferal tests and a standard limestone particle (NBS-19) are shown in Supplementary Fig. S2. The following nonlinear regression equation was used to calculate the bulk density of final chambers calcite in this study.

$$\begin{array}{rcl}{\rm{Bulk}}\,{\rm{density}}\,({\rm{g}}/{{\rm{cm}}}^{3}) & = & (\,-\,1.63\times {10}^{-5}){({\rm{Mean}}{\rm{CT}}{\rm{number}})}^{2}\\  &  & +\,(3.44\times {10}^{-3})({\rm{Mean}}\,{\rm{CT}}\,{\rm{number}})\mbox{--}15.5:{{\rm{R}}}^{2}=0.99\end{array}$$

### SEM observations

The crystalline structures of test walls were observed with a scanning electron microscope (JSM-6390V, JEOL, Ltd., Tokyo, Japan) at the Geological Survey of Japan, National Institute of Advanced Industrial Science and Technology. After the XMCT system observations and test weight measurements, some tests were prepared for SEM by embedding them in a resin. A cross section of the test wall was then polished, and the polished cross section was coated with platinum.

### Ambient seawater characteristics

Measurements of selected characteristics of seawater (temperature, salinity, pH, total alkalinity, and concentrations of nitrate and chlorophyll-a) were made less than two hours before the plankton collections as a part of the Post–WOCE Hydrography project^22^. The accuracy and traceability of the measurements were based on Global Ocean Shipboard Hydrographic Investigation Program standards^23^. CO_2_calc software^24^ was used to calculate ambient seawater carbonate ions concentration, [CO_3_^2−^]. All water masses where *G. bulloides* tests were sampled were supersaturated with respect to calcite (ΩCa > 1).

## Supplementary information

Supplementary Information

Supplementary Tables
