# Supplementary material for: Sensitivity of planktic foraminiferal test bulk density to ocean acidification
Source: Sci Rep. 2019 Jul 5;9:9803. doi: 10.1038/s41598-019-46041-x (PMC6611884; doi:10.1038/s41598-019-46041-x)
Supplement: Supplementary file 1 — Supplementary Information [file 41598_2019_46041_MOESM1_ESM.pdf]

Supplementary Information

Sensitivity of planktic foraminiferal test bulk density to ocean acidification

S. Iwasaki*,[1], K. Kimoto[1], O. Sasaki[2], H. Kano[2] & H. Uchida[1]

[1]Research Institute for Global Change, JAMSTEC, 2-15 Natsushima-cho, Yokosuka 237-0061, Japan. [2]The Tohoku University Museum, Tohoku University, 6-3 Aoba, Aramaki, Aoba-ku, Sendai 980-8578, Japan. *Correspondence and requests for materials should be addressed to S. I. (email: iwasaki.shinya@jamstec.go.jp)

Supplementary Figure S1
Supplementary Figure S2
Supplementary Figure S3
Supplementary Figure S4
Supplementary Figure S5
Supplementary Table 1
Supplementary Table 2

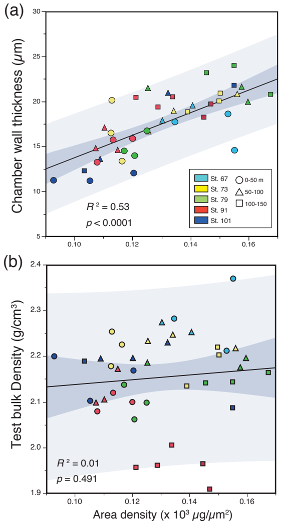

Supplementary Figure S1 | Plots of (a) outermost chamber wall thickness (μm) and (b) test bulk density (g/cm^3) of individual tests against the area density ($\mu g/\mu m^2$) observed in this study. The coefficient of determination (R^2), p-value, regression line, and the 95% confidence interval (dark shading) and prediction interval (light shading) are shown in each plot.

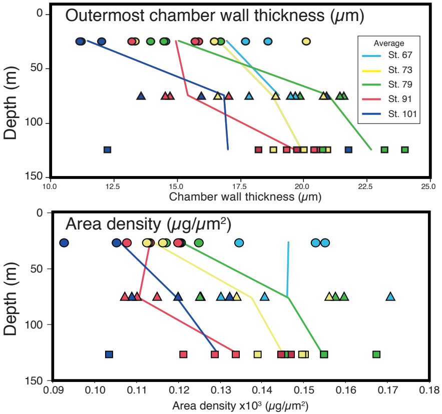

Supplementary Figure S2 : Change of outermost chamber wall thickness (μm) and the area density (μg/cm²) of *G. bulloides* tests along with the sampling depth at each sampling sites (m).

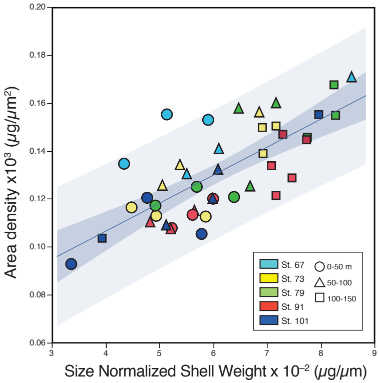

Supplementary Figure S3: Relationship between the test area density (µg/µm^2) and Size Normalize Shell Weight (µg/µm) at each sampling site and depth.

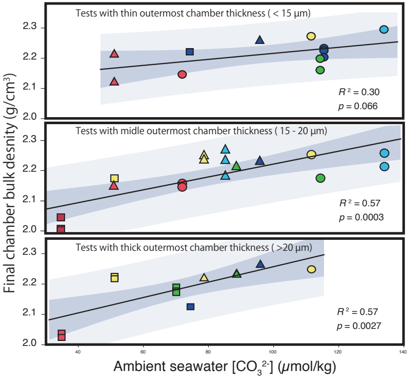

Supplementary Figure S4

Plots of final chamber bulk density (g/cm^3) against ambient seawater [CO_3^{2-}] where test were sampled under the three divisions of outermost chamber wall thickness. Thickness of outermost chamber wall is devided into three divisions; Thin: < 15 µm, Midle: 15 - 20 µm, Thick: > 20 µm. Final chamber bulk density of *G. bulloides* correlated with ambient seawater [CO_3^{2-}] in each division, suggestting that test bulk density of *G. bulloides* is controlled by ambient seawater [CO_3^{2-}] regardless of outermost chamber wall thicknening with growth.

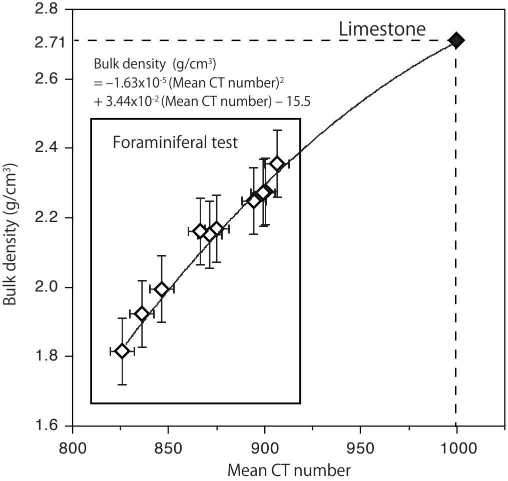

Supplementary Figure S2 | Plot of the relationship between mean CT number of foraminiferal tests measured by XMCT and the bulk density of the tests. The regression line fit to all the data, including the limestone data point, and the equation for estimating the bulk density of a foraminiferal test are also shown.

Supplemental Table 1. Locations, plankton tow sampling depth, and ambinet seawater characteristics

Station Number	Position		Depth (m)	Sample number	Temperature (°C)	Salinity (PSS-78)	Chlorophyll-a (μg/L)	$[NO_3^-]$ (μmol/kg)	$[CO_3^{2-}]$ (μmol/kg)
67 (KNOT)	44°05' N	154°59' E	0-50	3	3.5	33.1	0.59	0.3	134
			50-100	3	3.3	33.2	0.10	21.6	85
73 (K2)	47°01' N	160°01' E	0-50	3	3.2	32.7	0.69	12.4	112
			50-100	3	2.0	32.9	0.19	21.3	79
			100-150	3	3.6	33.3	0.03	34.2	51
79	46°59' N	166°44' E	0-50	3	3.6	32.9	0.73	12.1	115
			50-100	3	3.1	32.9	0.19	22.0	89
			100-150	3	3.1	33.4	0.03	31.1	70
91	47°00' N	173°49' E	0-50	3	5.6	32.8	0.38	12.0	72
			50-100	3	3.0	32.9	0.32	19.1	51
			100-150	5	2.6	33.1	0.03	27.5	35
101	47°00' N	174°57' W	0-50	3	5.9	32.9	0.39	12.5	116
			50-100	3	5.8	33.0	0.38	16.7	96
			100-150	2	5.4	33.4	0.04	25.6	75

Supplemental Table 2. Multiple regression analysis results

	Estimate	Std Error	t-value	p-value
**Test bulk density (Final chamber)**				
Intercept	5.81	1.95	2.98	0.005
Temperature	0.00	0.01	0.04	0.965
Salinity	-0.12	0.06	-2.00	0.053
Chlo a	-0.11	0.08	-1.48	0.148
$[NO_3^-]$	0.004	0.002	2.10	0.043*
$[CO_3^{2-}]$	0.003	0.000	6.34	<.0001*
**Wall thickness**				
Intercept	-19.7	33.4	-0.59	0.559
Temperature	-0.3	0.1	-2.27	0.029*
Salinity	0.8	1.0	0.79	0.433
Chlo a	-1.6	1.3	-1.18	0.246
$[NO_3^-]$	0.00	0.03	0.09	0.933
$[CO_3^{2-}]$	0.01	0.01	0.86	0.396
**Area density**				
Intercept	-1.1794	0.5557	-2.12	0.041
Temperature	-0.0073	0.0019	-3.91	0.0004*
Salinity	0.0401	0.0169	2.37	0.0233*
Chlo a	-0.0397	0.0219	-1.82	0.077
$[NO_3^-]$	-0.0001	0.0006	-0.27	0.789
$[CO_3^{2-}]$	0.0003	0.0001	2.40	0.0217*
**Mode of high CT number function**				
Intercept	908.9995	1305.1577	0.70	0.491
Temperature	-3.4264	4.3800	-0.78	0.4390
Salinity	-0.1767	39.7760	0.00	0.997
Chlo a	4.1227	51.3595	0.08	0.937
$[NO_3^-]$	1.2141	1.2946	0.94	0.354
$[CO_3^{2-}]$	0.8922	0.3318	2.69	0.0107*
